# Review of Polymer Drug Therapy for Cancer Driven by Artificial Intelligence

**DOI:** 10.3390/polym18060677

**Published:** 2026-03-11

**Authors:** Jie Zheng, Yuanlv Ye

**Affiliations:** 1 China Electronic Product Reliability and Environment Test Research Institute, Guangzhou 510610, China; 18620906209@163.com; 2 The College of Chemistry and Chemical Engineering, Lanzhou University, Lanzhou 730000, China; 3 Nuclear and Radiation Safety Center, Ministry of Ecology and Environment, Beijing 100084, China

**Keywords:** artificial intelligence (AI), polymer-based drugs, cancer therapy, drug delivery systems, tumor microenvironment (TME)

## Abstract

This review systematically evaluates the interdisciplinary convergence of artificial intelligence (AI) and polymer science in cancer therapy. Beyond mere description, we provide an integrated framework spanning synthetic optimization, biocompatibility prediction, and the design of tumor microenvironment (TME)-responsive carriers. We highlight how AI algorithms (ML, DL, and RNNs) transform traditional trial-and-error methods into a data-driven paradigm, enabling precise spatiotemporal drug release and individualized pharmacokinetic modeling. Crucially, this work addresses the critical gap between computational modeling and clinical realization by providing a balanced critical analysis of current bottlenecks, including the “small data” challenge, publication bias, and regulatory hurdles. We conclude with a roadmap for AI-guided precision oncology, shifting the focus from predictive accuracy to mechanistic interpretability and prospective in vivo validation.

## 1. Introduction

Cancer remains a major global public health challenge, driving an urgent need for precise diagnosis and efficient treatment [1,2]. However, conventional chemotherapeutic agents (e.g., cisplatin, doxorubicin) and imaging probes (e.g., indocyanine green) often suffer from poor targeting, low water solubility, suboptimal biocompatibility, and systemic toxicity, limiting their clinical efficacy [3,4,5,6,7]. To address these limitations, various nanodelivery platforms have been developed. However, existing platforms exhibit limitations: lipid nanoparticles are prone to physicochemical instability and premature drug release [8], while inorganic nanosystems face challenges including long-term bioaccumulation and imprecise drug release control [9,10]. In contrast, polymer-based delivery systems offer significant advantages, such as excellent stability, good biocompatibility, and highly controllable drug release profiles. By loading therapeutic or imaging agents onto polymer carriers, precise targeting and deep tissue penetration can be achieved, providing a crucial strategy to enhance cancer treatment efficacy.

As a core strategy, polymer-based sustained-release systems utilize biodegradable and biocompatible materials (e.g., FDA-approved PLGA) to encapsulate hydrophobic drugs. This approach not only enables controlled drug release but also significantly reduces systemic toxicity [11,12]. Beyond synthetic polymers, natural derivatives like chitosan and hyaluronic acid are widely used to construct functionalized carriers. Recently, stimulus-responsive intelligent systems that react to tumor microenvironment (TME) signals (e.g., acidic pH, elevated enzyme levels) have become a research focus. For example, PLGA nanoparticles can achieve pH-responsive drug release, while polyethylene glycol (PEG) modification prolongs the circulation half-life of formulations such as Doxil^®^ [13,14]. Redox-sensitive hyaluronic acid nanoparticles can reverse drug resistance in breast cancer, and chitosan-based systems enhance the efficacy of cisplatin and doxorubicin in non-small cell lung cancer through synergistic delivery. These systems have successfully overcome biological barriers, including the blood–brain barrier and abnormal vascularization in solid tumors.

The integration of artificial intelligence with polymer-based therapeutics is advancing the field toward precision and intelligent cancer treatment (Table 1). Machine learning algorithms can predict and optimize nanoparticle physicochemical parameters (e.g., size, surface charge) to maximize tumor accumulation [15]. For instance, AI models have guided the design of PLGA carriers to achieve drug-loading efficiency exceeding 80%, with dynamic release modulated by TME characteristics [16]. This synergy between polymer delivery systems and AI marks a new phase in oncology, moving toward predictable and programmable precision medicine. AI has also been applied in RNA therapy to optimize lipid–polymer hybrid materials, improving the stability and immunogenicity of cancer vaccines. The CURATE.AI platform exemplifies this by dynamically adjusting dosing regimens based on patient data to optimize the efficacy–toxicity balance [17].

AI further facilitates the integrated analysis of multi-omics data, guiding the design of targeted delivery systems to address tumor heterogeneity through genomic and proteomic insights [26]. By combining molecular dynamics simulations, AI enhances the understanding of nanocarrier–biological interface interactions, optimizing delivery strategies [27]. Machine learning platforms can accurately predict the in vivo distribution and metabolic fate of nanoparticles, providing a theoretical basis for tumor-specific delivery [28]. AI has also accelerated the development of long-acting polymer injectables by optimizing formulations through release curve prediction [29]. The convergence of these technologies heralds a new era of predictable and programmable precision medicine, offering novel solutions to overcome tumor heterogeneity and achieve personalized therapy. This review systematically elucidates how AI empowers the entire innovation chain of anticancer polymer materials, from conceptual design to functional realization.

## 2. Applications of Artificial Intelligence in Polymer Synthesis Design and Performance Prediction

In AI-driven polymer research, machine learning (ML) has emerged as a transformative strategy for accelerating material innovation, achieving significant progress in predicting polymer properties, designing structures, and optimizing synthesis conditions.

### 2.1. Utilizing Machine Learning to Optimize Polymer Synthesis Pathways

**Figure 1:**
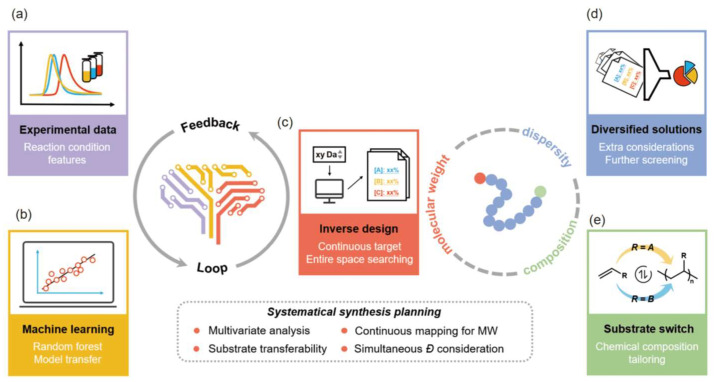
Operational workflow of the AI-driven polymer synthesis platform. The platform process is as follows: (**a**) Collect datasets and select appropriate inputs and outputs for research (e.g., initial feed ratio and desired molecular weight). (**b**) Screen and apply suitable machine learning algorithms to analyze datasets, establishing relationships between conditions and outcomes. (**c**) Traverse the entire condition space, using the model to derive synthetic instructions from target molecular weight results. (**d**) Screen the diverse output solutions to identify the optimal conditions that meet the Ð requirements. (**e**) Incorporate different reaction substrates into the platform using transfer functions, enabling customization for polymers with varying chemical structures. (**a**–**c**) form feedback loops for model establishment, while (**c**–**e**) describe three-dimensional customization of polymer synthesis. Reprinted with permission from Ref. [30]. Copyright 2021, Springer Nature.

**Figure 2:**
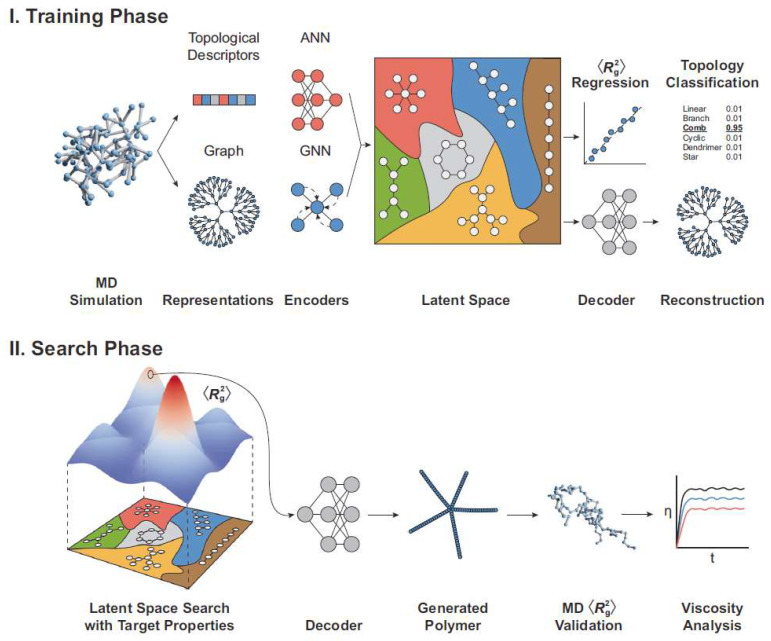
This diagram illustrates the fundamental strategy of the polymer topological variational autoencoder. During the training phase, molecular dynamics (MD) simulations are employed to compute computationally manageable descriptors, such as the mean square root radius (R^2^_g_) of a polymer ensemble. Subsequently, artificial neural networks (ANNs) and graph neural networks (GNNs) encode topological descriptors and polymer graph information into a low-dimensional latent space. Decoding of this latent space facilitates reconstruction, regression, and classification tasks. These encoded features are interconnected to form a reduced-dimensional latent space, from which the decoder reconstructs polymer structures. In the search phase, sampling points are drawn from the latent space to generate predictions for polymers exhibiting target R2g and specified topological structures. These predictions are evaluated using MD simulations, followed by systematic analysis of how topological structures influence other properties (e.g., viscosity) after validation. Reprinted with permission from Ref. [31]. Copyright 2024, Springer Nature.

By constructing algorithmic models such as neural networks and decision trees, researchers can predict polymer structures, molecular weights, and functional properties under various synthesis conditions (e.g., temperature, pressure, catalyst type, reactant ratios) [30,31]. This predictive capability provides precise guidance for experimental design, significantly reducing trial-and-error attempts, resource consumption, and development cycles (Figure 1). For instance, deep learning is widely applied in analyzing compound structures, screening compounds, and predicting synthetic routes, offering novel methods for chemical research (Figure 2). Applying AI to optimize synthetic pathways enables the design of polymer nanocarriers with customized dimensions, shapes, and surface characteristics to meet diverse therapeutic needs. AI platforms (e.g., TuNa-AI) have increased nanoparticle preparation success rates by 42.9% through automated formulation processes [32]. These innovations streamline R&D workflows and support combination therapies, such as co-encapsulating chemotherapeutic agents and immunomodulators for synergistic effects against heterogeneous tumors.

**Figure 3:**
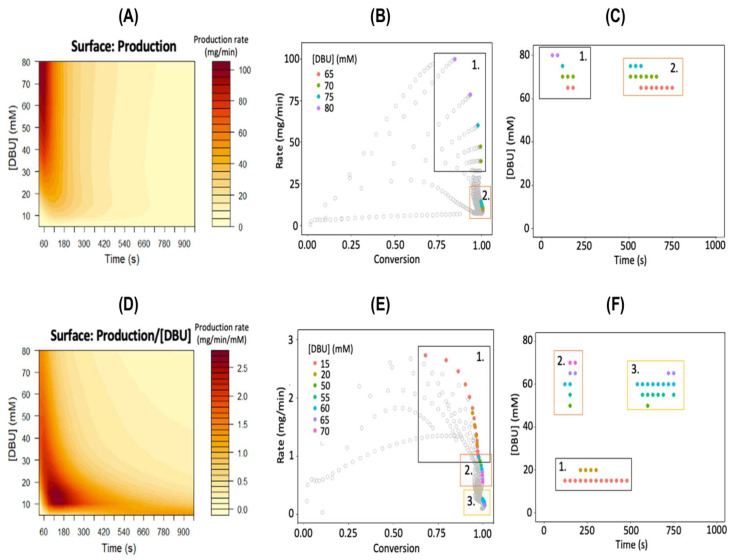
AI-assisted continuous flow synthesis of debutanol. (**A**) Isocurve plot showing production rate (unit: mg/min) as a function of residence time and debutanol concentration; (**B**) Pareto plot illustrating the relationship between production rate and conversion rate; (**C**) distribution of Pareto effective points on the [debutanol] vs. time axis; (**D**) isocurve plot of unit catalyst production rate (unit: mg/min/mmol) as a function of residence time and debutanol concentration; (**E**) Pareto plot of unit catalyst production rate versus conversion rate; (**F**) distribution of Pareto effective points on the [debutanol] vs. time axis. Note that each point in the Pareto plot represents the predicted conversion rate and rate corresponding to a specific compound-residence time combination. Colored dots in the figure indicate high-efficiency conditions during synthesis. Numbers 1–3 indicate the optimal process parameter range. Reprinted from Ref. [19].

Recent studies demonstrate that ML models achieve high precision in chemical reaction prediction by analyzing extensive reaction databases. For example, the Local Mapper model achieves 98.5% accuracy in predicting precise atomic mapping for organic reactions. In one study, an ML framework processed over 10,000 chemical reaction records and predicted polymer molecular weight distributions under specific conditions with over 90% accuracy rate exceeding 90% [19]. This enhances synthetic efficiency and facilitates the development of polymers with customized functionalities, such as improved mechanical strength or stimulus-responsiveness, crucial for biomedical and sustainable material applications. Furthermore, ML-driven optimization has been extended to autonomous synthesis platforms, where algorithm-integrated robotic systems enable real-time parameter tuning, advancing green manufacturing and reducing [33]. Recent advancements incorporate multi-objective optimization techniques (e.g., Pareto frontier) to balance competing objectives, as demonstrated in the organocatalytic ring-opening polymerization of L-lactic acid [19]. For an intuitive visualization, refer to Figure 3, which illustrates the functional relationship between production rate and variations in residence time and catalyst concentration [19].

### 2.2. AI-Predicted Biocompatibility of Polymer Materials

In AI-driven polymer research, predicting biocompatibility is a crucial step for biomedical applications, particularly in tumor therapy, where AI models accelerate the evaluation of biological interactions. With ML algorithms, scientists can rapidly and accurately assess polymer–biological system interactions, determining key indicators like cytotoxicity, immune response, and tissue integration without extensive wet-lab experiments. Deep learning models excel at deciphering complex relationships between polymer chemical structures (e.g., monomer composition, chain length, functional groups) and biocompatibility parameters.

**Figure 4:**
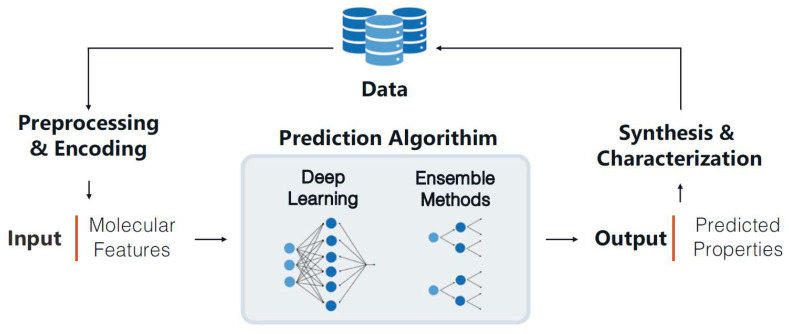
This diagram illustrates the general machine learning workflow for attribute prediction tasks. Data (i.e., polymers with known attributes) undergoes preprocessing and encoding (e.g., chemical structure encoding, molecular descriptor generation) prior to input into the prediction algorithm. Regardless of the algorithm selected, the training process minimizes prediction errors by adjusting model parameters. Reprinted with permission from Ref. [34]. Copyright 2023, Springer Nature.

**Figure 5:**
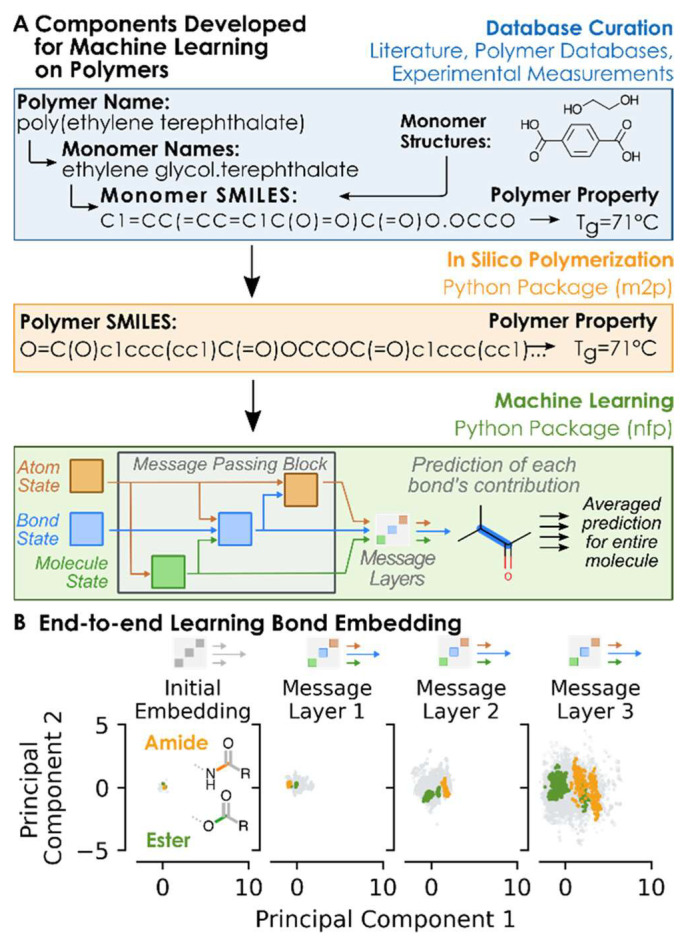
Systematic workflow and bond-feature discrimination of the PolyID. (**A**) The machine learning toolkit comprises: a training dataset mapping monomer structures to polymer properties, computer-simulated polymerization schemes, and a message-passing neural network for predictions. (**B**) As polymer structures traverse the layers, the principal component analysis of bond feature vectors demonstrates a significant enhancement in bond discrimination during message-passing. Acidamide bonds, ester bonds, and all other bonds are represented in green, orange, and gray respectively. Reprinted from Ref. [36].

For instance, a research team developed a comprehensive database containing tens of thousands of polymer structures and their biocompatibility data. By training convolutional neural networks to predict the biocompatibility of novel polymers, they achieved over 90% accuracy [34]. This high-precision tool significantly shortens material screening cycles, reduces costs, and mitigates risks, offering a rapid iterative solution for designing drug delivery carriers or implantable devices. The AI framework not only predicts but also optimizes models by integrating multi-omics data and quantum chemical simulations. Transfer learning and synthetic data generation have effectively addressed data scarcity issues [35]. These achievements are particularly significant in oncology, where highly biocompatible polymers must ensure safe targeted drug release while minimizing inflammatory or immune rejection risks. As shown in Figure 4, this workflow outlines the general ML process for property prediction, including chemical structure encoding and model training [34]. The schematic highlights the iterative process of data preprocessing, model optimization, and virtual screening, demonstrating AI’s role in bridging structural features and biological outcomes. Emerging AI tools like PolyID (Figure 5) can predict polymer properties from molecular structures, screening millions of candidates to identify biocompatible formulations for therapeutic applications [36].

### 2.3. Basic Functions and Targeting Strategies of Polymer Carriers

**Figure 6:**
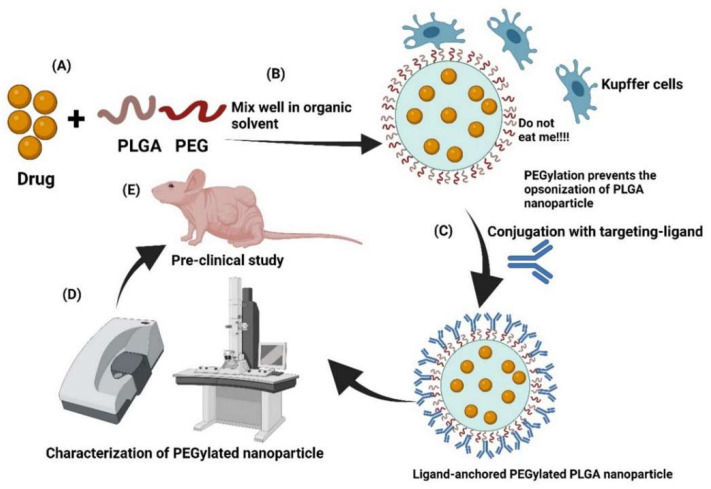
Schematic diagram of the preparation, targeting-ligand anchoring, characterization, and administration of PEGylated nanoparticles in tumor-bearing mice. (**A**) Drug conjugation with PEG; (**B**) Dissolution in organic solvent; (**C**) Conjugation with targeting ligands; (**D**) Characterization of nanostructure; (**E**) Preclinical validation. Reprinted from Ref. [16].

In cancer therapy, polymer nanocarriers show significant potential, particularly in drug delivery systems. PEG-conjugated nanoparticles exemplify this: they prolong drug circulation time and reduce immune clearance, significantly enhancing tumor accumulation [18]. Studies show PEGylated nanoparticles can extend drug half-life from minutes to hours [16]. As shown in Figure 6, this stealth effect stems from the hydrophilic PEG corona, which reduces macrophage phagocytosis, enabling passive tumor targeting via the enhanced permeability and retention (EPR) effect.

**Figure 7:**
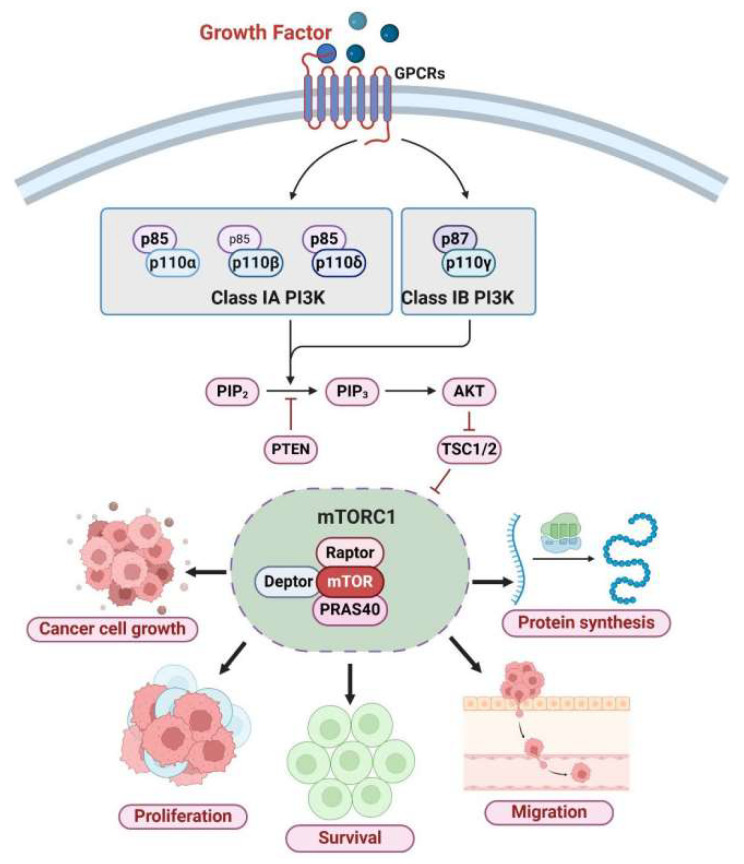
PI3K/AKT/mTOR pathway in cancer. When growth factors bind to their receptors, this pathway activates Class I phosphoinositide 3-kinase (PI3K). Receptor tyrosine kinases activate Class I PI3K, while G protein-coupled receptors activate Class I B. These two types of kinases convert PIP2 to PIP3, which recruits and activates AKT on the plasma membrane. Activated AKT phosphorylates and inhibits the negative regulators of mTORC1, TSC1/2. This inhibitory effect activates mTORC1—the primary regulator of protein synthesis, cellular metabolism, and growth. The mTORC1 complex promotes translation, driving cancer growth, proliferation, and survival. This mechanism also facilitates cancer migration and metastasis. Reprinted from Ref. [37].

Beyond basic encapsulation, biodegradable polymers like polylactic-co-glycolic acid (PLGA) offer tunable degradation rates and release kinetics, ideal for sustained or stimulus-responsive delivery. For example, in colorectal cancer models, drug-loaded PEGylated PLGA nanoparticles exhibit pH-sensitive release, leveraging the acidic TME to trigger release, increasing reactive oxygen species levels and inhibiting tumor proliferation [16]. In ovarian cancer treatment, hyaluronic acid-conjugated PEG-PLGA systems enhance cellular uptake by targeting CD44 receptors, effectively killing drug-resistant cell lines [37]. As shown in Figure 7, recent advancements include multifunctional designs like magnetically guided PEGylated nanoniosomes for co-delivering doxorubicin and curcumin, enabling precise targeting to accelerate therapy and reduce off-target effects [38].

To further enhance specificity, polymers play a pivotal role in targeted therapy. By covalently attaching targeting ligands (e.g., antibodies, peptides) to their surfaces, polymer nanocarriers can specifically recognize overexpressed receptors on tumor cells, enabling active targeting. In a breast cancer study, modified PEGylated polymer carriers successfully delivered drugs to tumors, showing significantly reduced systemic toxicity and improved efficacy compared to conventional chemotherapy [12]. Artificial intelligence is indispensable here. Deep learning models analyze vast biomolecular data to predict polymer-tumor cell interactions, guiding the synthesis of carriers with enhanced targeting. The latest platforms integrate AI with high-throughput screening to optimize lipid-polymer compositions, reducing toxic excipients by up to 75% while maintaining antitumor efficacy [32].

**Figure 8:**
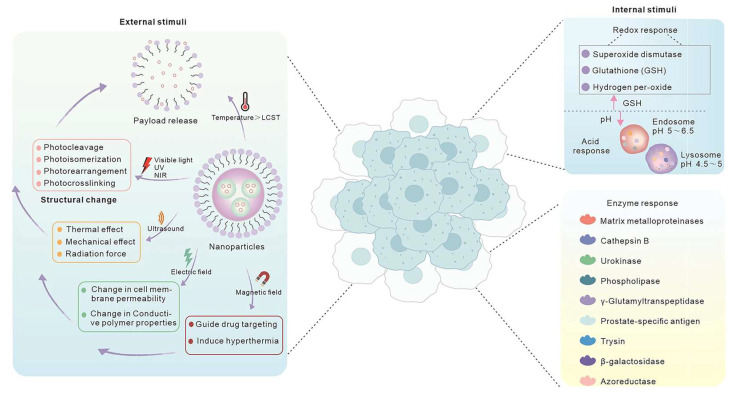
Classification of endogenous and exogenous stimuli for intelligent nanocarrier activation. The schematic differentiates between internal biological triggers (e.g., pH, enzymes, redox) and external physical triggers (e.g., light, heat, ultrasound, magnetic fields) used to achieve spatiotemporal control of drug release. Reprinted with permission from Ref. [39]. Copyright 2023, Springer Nature.

Furthermore, the evolution of TME-responsive polymers leverages unique tumor tissue features (e.g., pH gradients, enzyme activity) to design intelligent materials that release drugs under precise conditions. This ensures release primarily at the tumor site, minimizing damage to healthy tissues. In brain cancer, transferrin-modified PEG polymers enhance blood–brain barrier permeability, delivering siRNA to inhibit glioblastoma progression [39]. In summary, with AI, polymer materials not only improve treatment precision and efficiency but also pave the way for future intelligent cancer therapies. As shown in Figure 8, endogenous and exogenous stimuli (including pH, enzymes, redox signals) trigger responses in TME-responsive nanoparticles. Recent advances in immunotherapy, targeted therapy, and gene editing indicate a paradigm shift toward safer and more effective approaches.

## 3. Chemical Design and Mechanism of TME-Responsive Polymer Drugs

**Figure 9:**
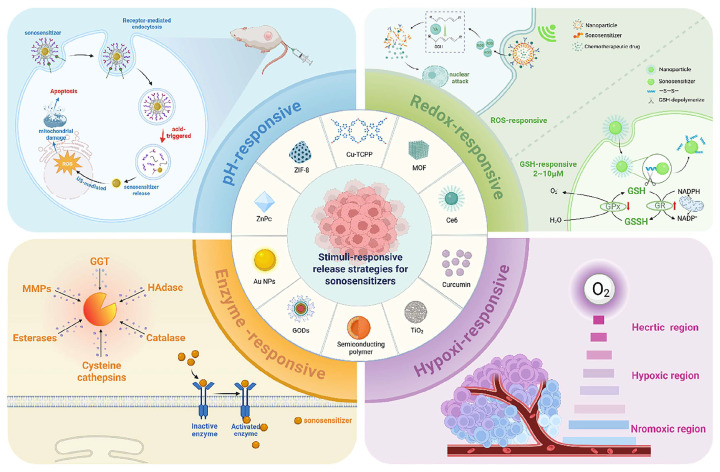
Adaptive drug release strategies triggered by tumor microenvironment (TME) stressors. This schematic illustrates the logic of stimuli-responsive systems that utilize physical and chemical stress signals (e.g., Enzyme, oxidative, Redox, or acidic stress) as endogenous triggers for payload liberation. Reprinted with permission from Ref. [40]. Copyright 2025, John Wiley and Sons.

The tumor microenvironment (TME) differs significantly from normal tissues in multiple biochemical indicators, providing natural triggers for designing intelligent polymer delivery systems that respond and release drugs under precise conditions. TME-responsive intelligent polymers have become a cutting-edge research focus. These materials are engineered to respond to specific TME conditions (as shown in Figure 9, e.g., low pH, high enzyme concentrations, elevated ROS, or glutathione levels), achieving targeted drug release at the tumor site while sparing healthy tissues.

AI has further revolutionized this field by employing deep learning for virtual screening of polymer libraries, predicting TME interactions via molecular dynamics simulations, and optimizing synthesis parameters [41]. For instance, generative adversarial networks (GANs) have designed novel hyperbranched polymers that adapt to dynamic TME changes, enhancing bioavailability and reducing off-target effects in preclinical models [42]. These breakthroughs are extending into clinical translation. In Phase II breast cancer trials, PEGylated pH-sensitive micelles show significant potential: AI-driven protocols can customize polymer formulations based on patient-specific TME characteristics [43]. This innovative design heralds a new era of precision oncology, minimizing systemic toxicity while markedly enhancing efficacy.

### 3.1. pH-Responsive Mechanism of Polymer Materials

The TME is typically more acidic (extracellular pH ~ 6.5–7.0, as low as 6.0) than normal tissues (near neutral pH) due to tumor cell metabolic alterations like glycolysis and lactate accumulation. This acidity is exploited to develop pH-sensitive polymer carriers that undergo structural changes (e.g., swelling, dissociation, bond cleavage) under acidic conditions, enabling controlled drug release. Researchers have developed polymers containing acid-labile bonds (e.g., hydrazones, acetals) or ionizable groups (e.g., carboxyl, amino groups). For example, certain nanoparticles rapidly disintegrate at pH < 7, releasing encapsulated drugs to directly attack tumor cells, enhancing efficacy while reducing cytotoxicity to normal cells [22]. Common structures include micelles with acid-labile bonds and polyhistidine derivatives that transition from hydrophobic to hydrophilic states, facilitating endosomal release.

**Figure 10:**
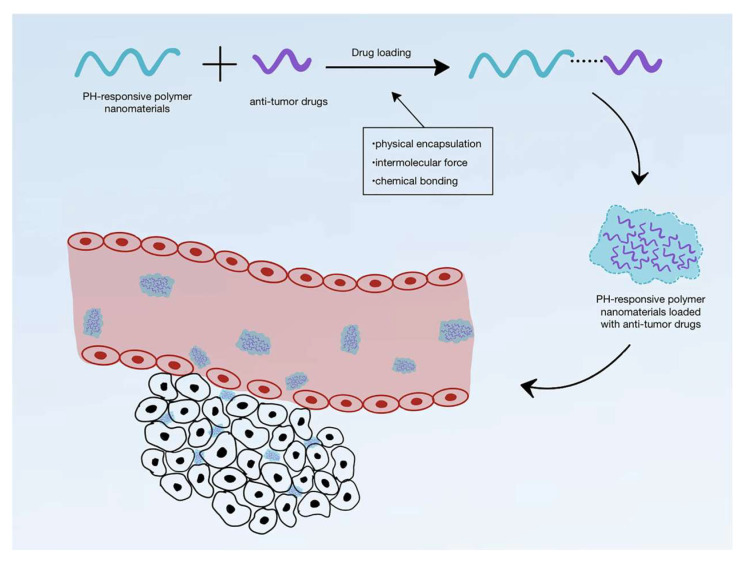
Schematic diagram of pH-responsive polymer nanomaterials loaded with antitumor drugs entering the tumor microenvironment. Reprinted from Ref. [22].

With AI, ML algorithms can predict and optimize polymer pH-sensitivity to ensure peak release under specific pH conditions. Neural networks trained on quantum chemical data can predict protonation states and conformational changes, simplifying design iterations and reducing experimental costs [13]. In clinical practice, AI-optimized pH-responsive hydrogels have been used in combination therapies to co-administer chemo- and immunotherapeutic agents, where acidic TME triggers sequential release to overcome resistance [14]. Recent studies highlight hollow molecularly imprinted polymers for DOX delivery, showing enhanced uptake in acidic environments and superior tumor suppression in vivo [13]. As shown in Figure 10, the pH-responsive mechanism improves drug delivery efficacy at tumor sites. Current research focuses on developing biodegradable variants for long-term safety, positioning such materials as core components of next-generation cancer therapies.

### 3.2. ROS-Responsive Mechanism of Polymer Materials

**Figure 11:**
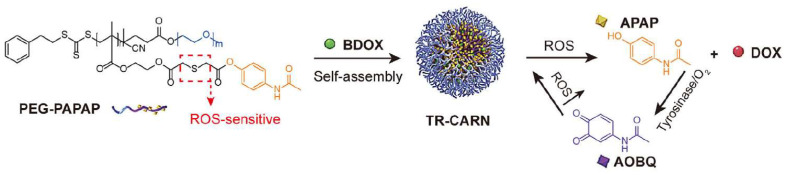
Schematic diagram of ROS-responsive prodrug preparation and cascade amplification release. Reprinted with permission from Ref. [46]. Copyright 2021, Royal Society of Chemistry.

Reactive oxygen species (ROS) levels within tumor cells (up to 50–100 μM) are significantly higher than in normal cells, providing an ideal trigger for ROS-responsive polymer platforms [44]. As shown in Figure 11, the core mechanism involves ROS-sensitive chemical bonds that undergo changes in chemical properties or cleavage upon oxidation at the tumor site, leading to carrier disassembly and drug release. Su et al. utilized this to design a star-shaped polymer achieving simultaneous ROS-triggered glutathione depletion, photodynamic therapy, and chemotherapy, demonstrating synergistic potential [45]. ROS-responsive platforms leverage tumor-specific oxidative stress for targeted and controlled release, representing a key strategy to enhance efficacy and reduce adverse effects.

### 3.3. Hypoxia-Responsive Mechanisms of Polymer Materials

**Figure 12:**
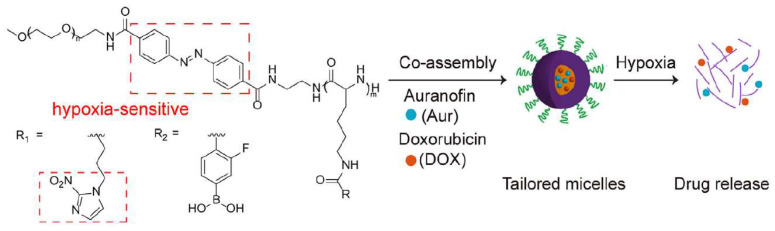
Schematic of hypoxia-responsive hybrid micelles for the co-delivery of Auranofin (Aur) and Doxorubicin (DOX). Reprinted with permission from Ref. [48]. Copyright 2025, Elsevier.

Solid tumors often contain hypoxic regions (pO_2_ < 10 mmHg) due to abnormal vasculature and rapid cell proliferation, providing specific targets for hypoxia-responsive polymer platforms [23]. As shown in Figure 12, such platforms primarily utilize hypoxia-sensitive groups (e.g., azobenzene, nitroimidazole) that are biologically reduced by intracellular reductases (e.g., azoreductase) under hypoxic conditions, triggering chemical structural changes and drug release.

To achieve precise control and overcome single-response limitations, advanced multi-responsive systems are being developed. For example, Guo et al. designed dual hypoxia-sensitive micelles (DHM) incorporating azobenzene (in the PEG corona) and nitroimidazole (in the hydrophobic core), enabling a two-stage “penetration first, release later” mechanism: azobenzene cleavage in hypoxic TME detaches the PEG corona, enhancing nanoparticle penetration; subsequent nitroimidazole reduction triggers micelle dissociation and drug release, significantly improving delivery to deep tumor tissues [47]. Gao et al. combined this with metabolic interference therapy, constructing a hybrid micelle system co-delivering doxorubicin and indomethacin. This platform achieved >90% tumor suppression in triple-negative breast cancer models by triggering drug release under hypoxia, where nitroimidazole reduction consumes NADPH, inhibiting tumor antioxidant defenses and synergizing with chemotherapy [48]. These studies demonstrate that intelligent polymer platforms leveraging tumor hypoxia can effectively address poor drug delivery and efficacy in hypoxic regions, offering novel insights for treating aggressive tumors when combined with immunomodulatory and metabolic strategies.

### 3.4. Enzyme-Responsive Mechanisms of Polymer Materials

Overexpression of various enzymes (e.g., matrix metalloproteinases (MMPs), caspase-1 (CTSB), esterases) in the TME provides highly specific biological signals for enzyme-responsive polymer platforms [49]. The core design strategy involves covalently linking drugs or imaging agents to the polymer backbone via enzyme-substrate sequences (e.g., specific peptides) or cleavable bonds (e.g., esters). When enriched at the tumor site, carriers are specifically cleaved by overexpressed enzymes, achieving precise controlled release and avoiding premature leakage or off-target toxicity.

**Figure 13:**
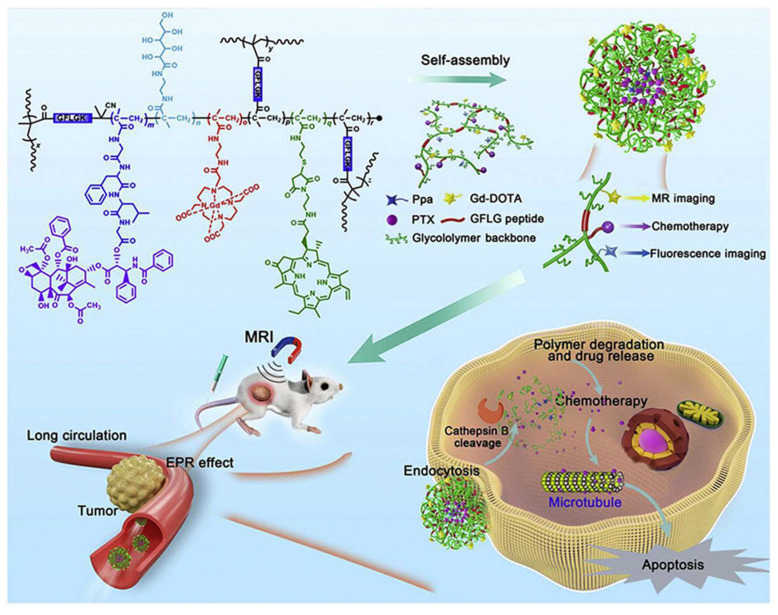
Schematic of Cathepsin B-responsive biodegradable branched glyco-polymers. Reprinted from Ref. [49].

As shown in Figure 13, under protease CTSB action, the polymer scaffold degrades to release PTX while activating imaging signals, enabling real-time therapy monitoring. This approach achieved a 90.6% tumor growth inhibition rate in a breast cancer model [49]. Enzyme-responsive platforms, due to their high biological specificity, minimize off-target toxicity, making them promising for precision oncology and theranostics. By selecting enzymes and substrates specific to particular tumor types, personalized, efficient, and low-toxicity therapies can be realized.

### 3.5. Multi-Responsive Mechanism of Polymer Materials

**Figure 14:**
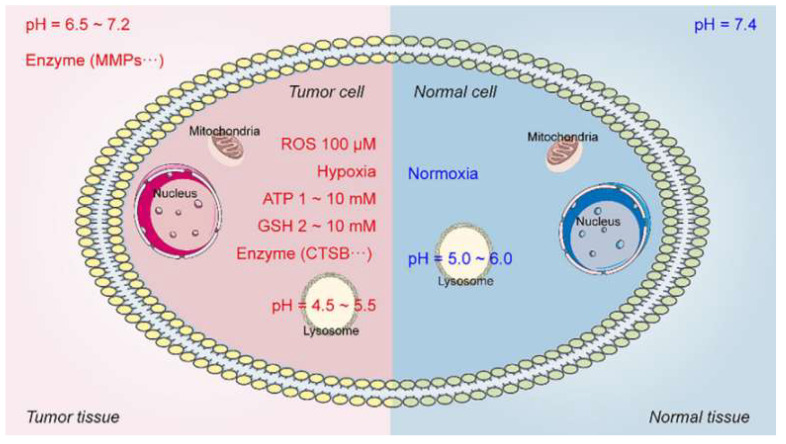
Comparative biochemical landscapes of normal versus tumor tissue microenvironments. ATP: adenosine triphosphate; CTSB: caspase B; GSH: glutathione; MMPs: matrix metalloproteinases; ROS: reactive oxygen species. Reprinted with permission from Ref. [51]. Copyright 2025, John Wiley and Sons.

**Figure 15:**
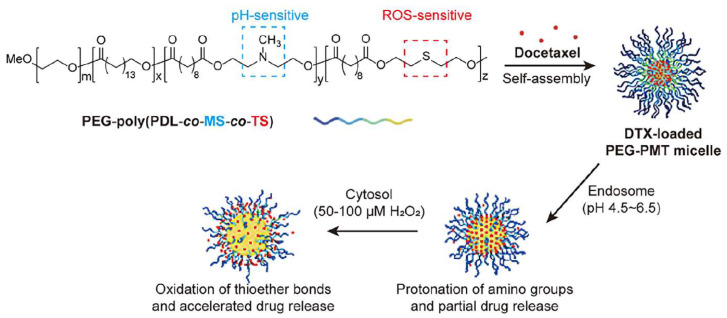
Design and mechanism of ROS/pH dual-responsive polymeric nanocarriers. This schematic depicts the programmed release of therapeutic agents triggered by the synergistic acidic and oxidative (ROS) signals within the tumor microenvironment (TME). Reprinted with permission from Ref. [52]. Copyright 2020, Elsevier.

Multi-responsive polymer materials demonstrate significant advantages in drug delivery. As shown in Figure 14, a recent study used an ML model to analyze extensive datasets of polymer structures and pH-responsive behaviors, designing a nanocarrier that initiates release at pH 6.5. This significantly enhances tumor drug concentration while reducing normal tissue toxicity [50]. Such intelligent mechanisms improve efficacy and minimize adverse effects, highlighting AI’s potential in polymer design. Beyond pH, enzyme-responsive polymers can utilize overexpressed MMPs in TME to cleave peptide chains, selectively releasing payloads like doxorubicin. Hypoxia-responsive systems containing nitroimidazole groups activate under low oxygen tension, addressing hypoxia-induced drug resistance in solid tumors [51]. Recent innovations involve highly stimuli-responsive polymers sensitive to both pH and ROS (Figure 15). These polymers, containing azobenzene or thioether groups, degrade under oxidative stress, synergizing with acidic signals to enhance specificity [52].

**Figure 16:**
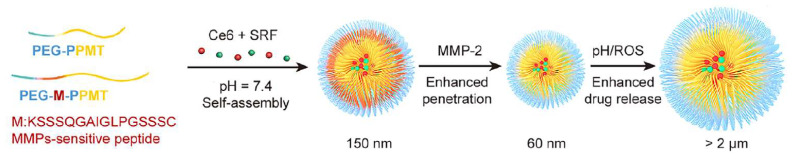
Design and triggered release mechanism of pH/ROS/enzyme triple-responsive nanocarriers. Schematic diagram of a pH/ROS/enzyme triple-responsive drug nanocarrier: PEG-M-PPMT nanoparticles loaded with SRF and Ce6. Reprinted with permission from Ref. [53]. Copyright 2021, Elsevier.

In mechanism design, dual-responsive systems combining pH with redox or enzyme triggers can achieve hierarchical release regulation. For example, glutathione/pH dual-sensitive nanocarriers disintegrate in the reductive TME, enhancing ROS-induced apoptosis [41]. Furthermore, Shu et al. developed a triple-responsive platform for MMP-2, pH, and ROS, co-delivering sorafenib and photosensitizer Ce6 for synergistic chemo-photothermal therapy (Figure 16) [53]. The release mechanism involves multi-level responses: MMP-2 cleaves surface PEG fragments in the extracellular matrix, enhancing penetration; upon entering cells, acidic pH and high ROS induce nanoparticle swelling and rapid drug release. Ce6 not only induces apoptosis but also amplifies ROS generation, accelerating drug release.

In summary, dual- and multi-responsive polymer platforms enhance targeting precision and release efficiency, enabling safer and more adaptable treatment strategies. They effectively address key challenges like tumor heterogeneity, penetration barriers, and off-target toxicity, transforming complex biochemical responses into controllable drug release behaviors and injecting new hope into cancer therapy.

## 4. AI-Assisted Precision Oncology Treatment Strategies

In precision oncology, AI is progressively transforming treatment paradigms by enabling data-driven customization of therapy, moving beyond traditional one-size-fits-all approaches. Through machine and deep learning, AI analyzes vast amounts of patient data, including multi-omics data (genomics, proteomics, metabolomics), to identify molecular tumor characteristics and predict treatment responses [32]. For instance, AI-powered prediction of biomaterial biocompatibility enables more precise drug delivery systems, ensuring safety and efficacy. In clinical practice, AI-assisted strategies can design customized regimens based on TME-responsive polymer properties, modulating drug release in response to tumor pH changes to enhance targeting. AI also shows significant potential in optimizing polymer drug pharmacokinetics; by predicting and simulating release processes, precise control of release rates can be achieved, further improving precision treatment outcomes.

### 4.1. Artificial Intelligence Optimization of Polymer Drug Release Kinetics

In cancer therapy, polymer drug release kinetics are a key determinant of efficacy. Optimizing this process with AI can significantly enhance targeting and efficiency. For example, by analyzing large experimental datasets with ML algorithms, researchers can predict release behavior under different pH or temperature conditions, designing smart polymers for stable release in TME. Studies show deep learning models can predict drug release curves with less than 5% error [21], providing robust support for preclinical research. Compared to traditional mechanistic models (e.g., Higuchi), AI provides superior predictive accuracy for complex, non-linear systems but lacks the same level of physical interpretability. Prospective in vivo validation remains a crucial missing link in the current literature [25,54].

**Figure 17:**
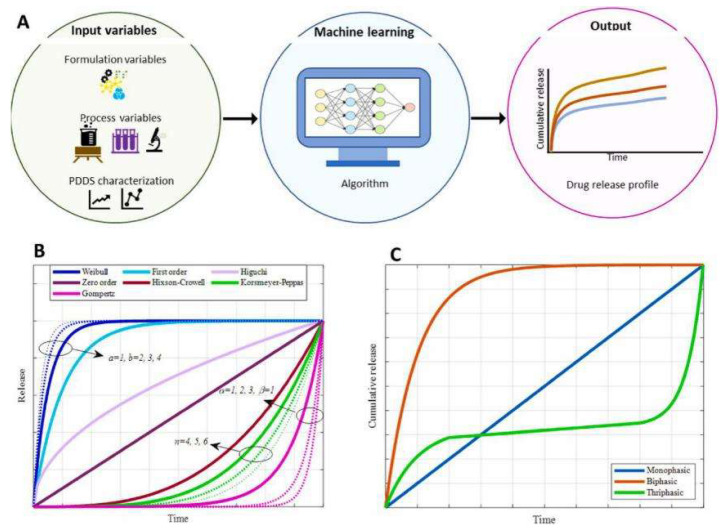
Integrated framework and comparative analysis for drug release modeling. (**A**) The main framework of machine learning in drug release modeling, (**B**) visualization of release curves from various mathematical models including Higuchi, Hixson–Crowell, Korsmeyer–Peppas, Weibull, and Gompertz, (**C**) typical in vitro drug release patterns of PDDS. Reprinted from Ref. [21].

Furthermore, AI optimizes release kinetics through precision treatment planning. By analyzing patient tumor characteristics and medical history, AI algorithms can develop customized release protocols to ensure optimal release rates and therapeutic outcomes. As shown in Figure 17, reinforcement learning models can simulate release processes for different patients, creating personalized release curves. This strategy enhances precision and reduces side effects. AI simplifies the construction and optimization of release kinetics models, marking a revolutionary breakthrough in cancer treatment.

### 4.2. Artificial Intelligence for Optimizing Pharmacokinetics and Efficacy Prediction

**Figure 18:**
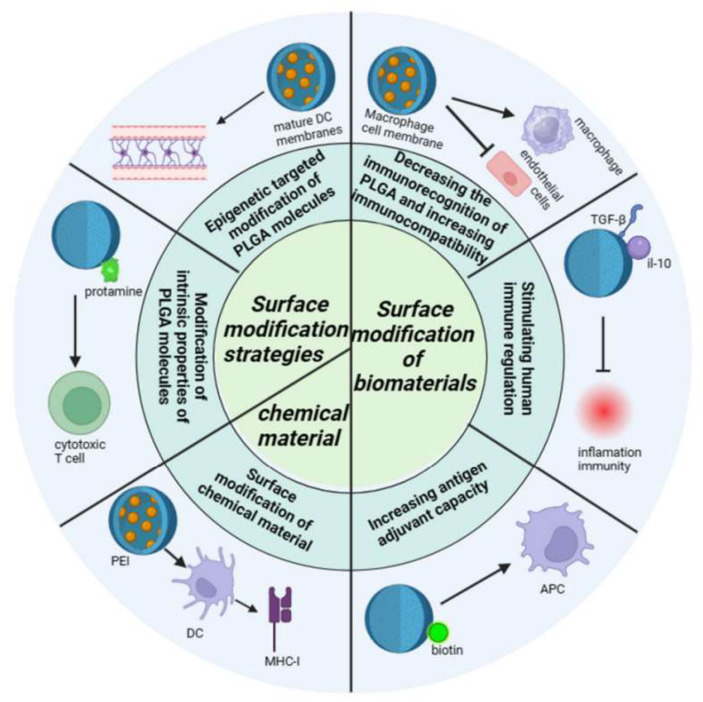
Modification mechanisms and immunomodulatory effects of PLGA-based systems. The modification mechanism of PLGA illustrates that PLGA interacts with immune cells after being modified by biomaterials and chemical materials, influencing inflammatory responses and immune effects, as well as the impact of modification strategies on the barrier penetration capability of PLGA; normal arrows indicate promotion or activation, while arrows with bars indicate inhibition. Reprinted from Ref. [20].

Biodegradable polymers offer significant advantages: they effectively deliver drugs to tumors and safely degrade post-mission, reducing long-term implantation risks. For instance, PLGA degrades into lactic and glycolic acid, normal metabolic byproducts with excellent biocompatibility. As shown in Figure 18, a study using PLGA nanoparticles to deliver doxorubicin significantly increased tumor drug concentration while reducing normal tissue toxicity [20].

### 4.3. Artificial Intelligence for Predicting Biodegradation of Polymer Materials

**Figure 19:**
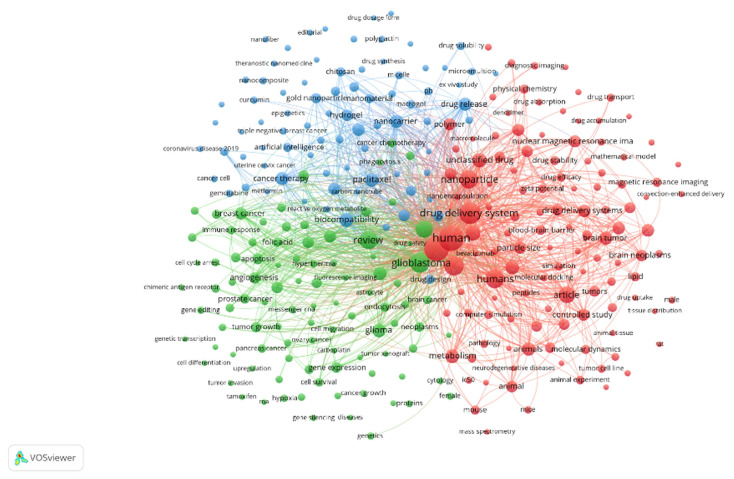
VOSViewer co-occurrence analysis. Reprinted from Ref. [55].

AI also plays a pivotal role in predicting and optimizing polymer biodegradation rates. Through ML models, researchers can accurately predict degradation behavior under specific conditions, guiding the design of clinically translatable delivery systems. In AI-driven polymer research, predicting biodegradation is core to precise tumor therapeutic design. Algorithms like support vector machines or deep learning networks can construct complex structure–activity relationship models between chemical structures and degradation kinetics (Figure 19). For example, deep learning models analyzing PLGA degradation accurately predicted rates under varying pH and enzyme concentrations [55]. This capability is crucial for designing TME-responsive polymers that must safely degrade after therapy. AI is turning this rational design into reality, promoting innovative applications in cancer therapy.

## 5. Summary and Outlook

In recent years, AI has emerged as a core driver for optimizing polymer synthesis and accelerating functional material development. By leveraging convolutional neural networks (CNNs) and support vector machines (SVMs), researchers can now accurately predict synthetic pathway feasibility and degradation kinetics, significantly reducing trial-and-error costs. In the realm of precision oncology, the integration of recurrent neural networks (RNNs) with multi-omics data—such as patient-specific genomic and proteomic profiles—has pushed polymer research into the era of personalized medicine. These intelligent strategies facilitate the rational design of next-generation nanocarriers capable of precise targeting and stimuli-responsive drug release, thereby maximizing therapeutic efficacy while minimizing systemic biosafety risks.

Despite these advancements, several critical bottlenecks hinder the seamless clinical translation of AI in polymer therapeutics. The “small data” problem remains a primary obstacle, as polymer datasets are often fragmented compared to other fields, making models prone to overfitting. Furthermore, a prevalent publication bias toward positive results leads AI models to “optimistically” overestimate success rates. The lack of standardized data formats and open-source model weights further limits reproducibility and cross-verification. Crucially, most current models lack external validation in independent, multi-center experimental trials, and the inherent “black-box” nature of deep learning poses significant regulatory challenges regarding the physical interpretability of polymer–drug interactions.

Looking ahead, the synergy between AI and polymer science is expected to expand into cutting-edge domains such as cancer immunotherapy and in vivo gene editing. Future research should prioritize the development of “Explainable AI” (XAI) to bridge the gap between high-accuracy predictions and mechanistic understanding. By establishing standardized, open-access databases and incorporating more prospective in vivo validation, the field can transition from purely computational modeling to robust, clinically applicable polymer platforms. These breakthroughs will enable the design of intelligent polymers that not only respond to the tumor microenvironment but also actively participate in modulating complex biological pathways.

In conclusion, the integration of AI into polymer materials science holds unprecedented transformative potential for cancer therapy. While technical and regulatory challenges regarding data quality and model interpretability persist, the shift toward an AI-driven, data-centric paradigm marks a revolutionary milestone. Overcoming these hurdles will ultimately pave the way for a new generation of precision nanomedicines, bringing significant breakthroughs to the future of oncology.

## Figures and Tables

**Table 1:** Comparative summary of AI methodologies in polymer-based drug delivery.

Methodology	Primary Applications	Input Features	Dataset Scale	Validation & Performance	Validation	Ref.
Machine Learning (ML)	Degradation & Biocompatibility prediction.	Molecular descriptors, SMILES fingerprints.	Small (10^2^–10^3^)	K-fold cross-validation; R^2^ > 0.85, RMSE ~ 10%.	Mostly In vitro	[18,19]
Deep Learning (DL)	Synthesis optimization; Multi-omics integration.	Molecular graphs, 3D grids, genomic data.	Medium/Large (10^3^–10^5^)	Internal split-sets; <5% error in curve fitting.	In vitro & In vivo	[20,21]
Generative Adversarial Networks (GANs)	De novo polymer design.	Structural templates, target properties.	Medium (10^3^–10^4^)	Validity/Novelty scores; >90% structural novelty.	In silico/In vitro	[22,23]
Reinforcement Learning (RL)	Dynamic release & Treatment planning.	TME states, dose–response history.	Simulation-driven	Reward convergence; Optimized therapeutic window.	Pre-clinical	[24,25]

## Data Availability

No new data were created or analyzed in this study. Data sharing is not applicable to this article.
